# A Wide-Range Displacement Sensor Based on Plastic Fiber Macro-Bend Coupling

**DOI:** 10.3390/s17010196

**Published:** 2017-01-20

**Authors:** Jia Liu, Yulong Hou, Huixin Zhang, Pinggang Jia, Shan Su, Guocheng Fang, Wenyi Liu, Jijun Xiong

**Affiliations:** 1 Key Laboratory of Instrumentation Science & Dynamic Measurement, Ministry of Education, North University of China, Taiyuan 030051, China; 18734920710@163.com (J.L.); zhanghx@nuc.edu.cn (H.Z.); pgjia@nuc.edu.cn (P.J.); 18880480379@163.com (S.S.); liu_wenyi418@126.com (W.L.); xiongjijun@nuc.edu.cn (J.X.); 2 Science and Technology on Electronic Test & Measurement Laboratory, North University of China, Taiyuan 030051, China; gcfang_nuc@163.com

**Keywords:** POF, macro-bend, coupling, displacement sensor

## Abstract

This paper proposes the strategy of fabricating an all fiber wide-range displacement sensor based on the macro-bend coupling effect which causes power transmission between two twisted bending plastic optical fibers (POF), where the coupling power changes with the bending radius of the fibers. For the sensor, a structure of two twisted plastic fibers is designed with the experimental platform that we constructed. The influence of external temperature and displacement speed shifts are reported. The displacement sensor performance is the sensor test at different temperatures and speeds. The sensor was found to be satisfactory at both room temperature and 70 °C when the displacement is up to 140 mm. The output power is approximately linear to a displacement of 110 mm–140 mm under room temperature and 2 mm/s speed at 19.805 nW/mm sensitivity and 0.12 mm resolution. The simple structure of the sensor makes it reliable for other applications and further utilizations, promising a bright future.

## 1. Introduction

Displacement is an important physical quantity of solid mechanics, and displacement measurements are required in a variety of applications, such as precision alignment, position monitoring, vibrations analysis and robotics [1,2,3]. Since a decade ago, a number of displacement measurement sensors, such as inductance, capacitance, ultrasonic and fiber-optic displacement sensors [4], have been developed. Their inductance type transducer exhibits a good linear response in the measurement and no electrical contact, as the displacement changes with the inductance. However, problems include: the low frequency response, coil heating, and electromagnetic attraction and large size [5]. Capacitance-type displacement sensors have high sensitivities, but the parasitic capacitance influences the measurement result [6]. Also, the inductance and capacitance displacement sensor increases the fabrication cost. 

Conversely, fiber-optic sensors retain many advantages compared with the aforementioned sensors, such as fast speed, electrical passivity, and immunity to electromagnetic interferences, and provide possibility for measurement of displacement. Currently, fiber-optic grating displacement sensors are more widely used, for example, Cantilever beam type fiber-optic grating displacement sensor with linear response of 0.058 nm/mm within a displacement range of 0–20 mm [7]. Jicheng Li et al. obtain sensor information via the external displacement parameters modulated the Bragg wave, and achieve measurement range of 550 mm with 14 pm/mm sensitivity and 0.142 mm accuracy, but the fabrication process is more complicated [8]. Compared with the fiber-optic grating displacement sensors, intensity-modulated sensors offer a simpler and lower cost approach to detect the displacement. Among intensity-modulated ones, the macro-bending system is easier to implement, showing a good potential for displacement applications. For example, Erik et al. [9] present a glove-based sensor based on the light intensity attenuation owing to the fact that fiber micro-bending losses are correlated to the variations in flexing angle, with a sensitivity of 1.80°, and can be suitable for applications in measurement angular displacements of a robot. Arifin et al. [10] scraped away the outer surface of the cladding to improve the sensitivity of the displacement measurements, and achieve maximum sensitivity of 0.2401 μW/mm and finest resolution of 4.2 μm, but the measurement range is only 0–15 mm. Based on the fiber fundamental core mode coupling and the Whispering Gallery modes (WGMs) induced by the reflection at the clad-coat interface, a length of acrylic jacket fiber has been stripped, then coated with absorbent coating material. This achieves a measurement displacement of 0–30 mm [11]. However, from the above literature, as optical fiber displacement sensors on the basis of macro-bend loss usually use a single fiber with treatment, there is no doubt that the treatment decreases the sensor robustness. Conversely, perfection plastic fiber in macro-bend, because of its advantage of flexibility, has a good robustness and shows good potential in the displacement sensing field.

In previous work, we have realized liquid level detection based on effect of the Cladding Mode Frustrated Total Internal Reflection (CMFTIR) in POF, with the extinction ratio of the liquid level probe of 4.18 dB [12]. This paper further proposes a wide-range displacement sensor, the strategy and fabrication of an all plastic fiber wide-range displacement sensor, by using the macro-bend coupling effect which causes power transmission and variation between two twisted bending plastic optical fibers. This structure has a good robustness and the fiber does not need pretreatment. Through the Beam Quality Analyzer analyzing the energy transmission from one fiber to another, it can be seen that the optical field distribution and the coupling power change with the bending radius of the two twisted fiber. The displacement sensor performance is tested at different temperatures and speeds and is found to be satisfactory at both room temperature and 70 °C when the displacement is up to 140 mm. The output power is approximately linear with displacement of 110 mm–140 mm under room temperature and 2 mm/s speed with 19.805 nW/mm sensitivity and 0.12 mm resolution. The retrace error of the system is less than 0.05 nW/mm. 

## 2. Sensor Design and Sensing Principles

**Figure 1:**
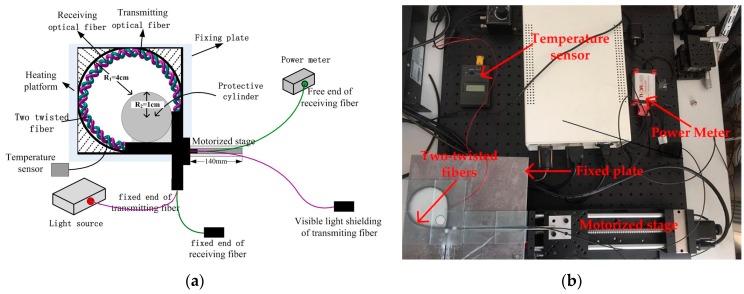
System of wide-range displacement sensor (**a**) and Displacement sensor experiment device (**b**).

The displacement sensor system is shown in Figure 1. It consists of an LED optical source (LEDD1B, Thorlabs, Newton, NJ, USA), a Power Meter (S120vc, Thorlabs), a fixed plate of the diameter of 8 cm, a guard cylinder with a diameter of 1 cm placed in the plate groove to prevent the bent radius of fibers that are too small to break the sensor when the fiber free end is moving. A temperature sensor monitored the temperature of fiber in real time. A macro-bend structure of two twisted naked plastic optical fibers in the plate groove works as the sensing element which is fixed on the Heating platform (MS-H280-Pro). One end of the two twisted optic fibers is the ‘fixed end’, and the other end, the ‘free end’. The free end of two twisted fibers is fixed on the motorized stage (Y200MC). The fixed end of receiving fiber is covered with a black hat to shield the visible light, and the free end of the fiber is connected to power meter. The fixed end of transmitting fiber is connected to optical source, and the other end is covered with a black hat to shield visible light. The Motorized stage is used to draw the free end of the two twisted fibers to achieve displacement shifts. 

In Figure 1, when the free ends of twisted structure move forward, the macro-bend radius R is changed simultaneously. It can be described as:

(1) $$R=\frac{d}{2\pi }+R_{2}$$

where d is the displacement of fiber free ends, R_2_ is the radius of the guard cylinder.

The fiber macro-bend radius is smaller than a certain threshold, which causes optical fiber mode field distortion, so that the light confined in fiber originally radiates outside of the fiber. The outside radiation generates a lot of macro-bend radiation modes and cladding modes, where macro-bend radiation modes mainly come from the refracted light rays and tunneling rays. Transmission coefficients of the refraction effect and the tunneling rays are expressed by Τ_r_ and Τ_t_, respectively [13], as

(2) $$T_{r}=\frac{4\sin\theta {\left(\sin^{2}\theta -\sin^{2}\theta_{c}\right)}^{1/2}}{{\left[\sin\theta +{\left(\sin^{2}\theta -\cos^{2}\theta_{c}\right)}^{1/2}\right]}^{2}}$$

(3) $$T_{t}=\frac{4\sin\theta }{\sin\theta_{c}}{\left(1-\frac{\sin^{2}\theta }{\sin^{2}\theta_{c}}\right)}^{1/2}\exp\left[-\frac{2}{3}n_{1}k\times \left(R+r\right){\left(\theta_{c}^{2}-\theta^{2}\right)}^{2/3}\right]$$

where, k=2π/λ is the free-space propagation constant, r is the radius of the cylindrical homogeneous core, n_1_ is the core index of fiber, θ is the angle between the light ray and the optical fiber axis, θ_c_ is the minimum critical angle for total internal reflection, when the light ray is propagating in fiber. The angle is described by Snell’law [14], as

(4) $$\theta_{c}=\arcsin\left(n_{2}/n_{1}\right)$$

n_2_ is the cladding index of fiber.

**Figure 2:**
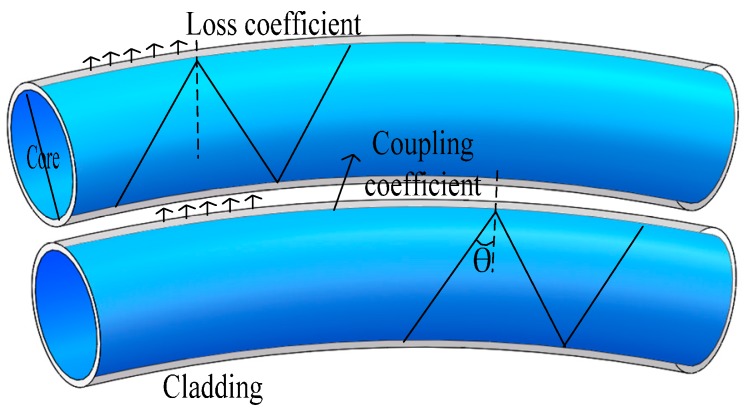
Macro-bend coupling effect between two fibers.

One part of transmitting fiber light radiates to the outside of fiber cladding when the fiber bends, and then forms a radiation field around at the space of cladding. An energy coupling effect occurs when the receiving fiber traverses close to the transmitting fiber enough and exposes it to a radiation field. Then, the radiation light couples and transmits in the receiving fiber, which results in a certain intensity coupling in receiving the fiber without original energy. This phenomenon is called the fiber macro-bending coupling effect (FMCE), with the transmission schematic shown in Figure 2. The two twisted structure enhances the coupling effect and stabilizes the coupling coefficient, so the two flexible plastic optical fibers with thin cladding are essential. Step-index fibers (Mitsubishi, SK-40) with thickness of 10 μm cladding and core diameter of 980 μm are adopted in this experiment; thus, the refractive index of the cladding and the core are 1.402 and 1.492, respectively. The calculation shows that Τ_t_ is very small and close to 10^−8^. It can be approximated that the macro-bending loss is caused by Τ_r_.

Actually, the energy coupling situation is very complex between the two closely bending fibers. When fiber macro-bending happens, more energy radiates outside of the transmitting fiber. Refracted at cladding-environment surface and coupling into receiving fiber, it radiates outside of the receiving fiber. However, this energy is ignored because it is smaller than the energy of the transmitting fiber coupled into receiving fiber. Therefore, only the energy of transmitting fiber coupling into the receiving fiber should require our attention. So, the coupling coefficient is C, and the energy coupled into the receiving fiber is expressed as:

(5) $$P_{1}=P_{0}\alpha_{1}C$$

where α_1_ is the loss coefficient of transmitting fiber.

At the same time, radiation occurs in the receiving fiber in a similar way. Because there are mainly higher order modes, the transmission loss cannot be casually equivalent to transmitting fiber loss. The loss coefficient of the higher-order modes for the step-index optical fiber is expressed as [15]:

(6) $$P_{0}=2n_{1}k(\theta_{c}^{2}-\theta^{2})\exp\left[-\frac{2}{3}n_{1}\mathrm{kR}{\left(\theta_{c}^{2}-\theta^{2}-\frac{2r}{R}\right)}^{3/2}\right]$$

Then output power at the free end of the receiving fiber can be expressed as:

(7) $$P_{2}=P_{1}\left(1-\alpha_{2}\right)=P_{0}\alpha_{1}C\;\left(1-\alpha_{2}\right)$$

where α_2_ is the loss coefficient of receiving fiber.

As can be seen from aforementioned equations, the energy coupling and transmission loss take place between two close fibers, and according to the calculation, the output power of the receiving fiber changes directly with the displacement, which realizes displacement sensing based on this principle.

## 3. Experiment and Results 

**Figure 3:**
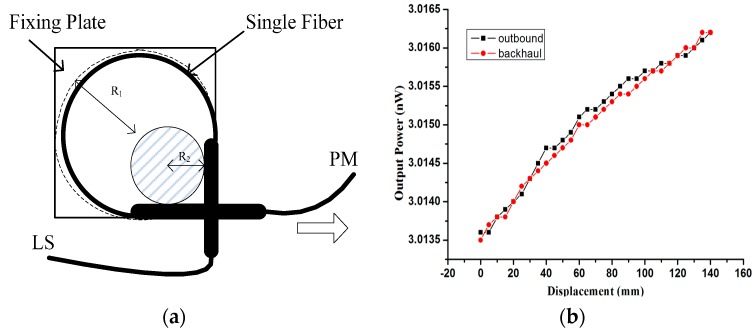
System of single fiber displacement sensor (**a**); Output power changes with displacement (**b**).

A single-fiber macro-bending structure for a displacement sensing system is shown conceptually in Figure 3a, with one end of the fiber connected to a Light Source (LS) and another end connected to a Power Meter (PM), all parameters are identical with Figure 1 except the light source is laser. The radius of the macro-bend decreases with the displacement increase, the output power changes become disorderly and unsystematic. Because the background noise of the transmitting fiber is mainly composed of core mode fluctuations, influenced by the light source fluctuations, it shows poor signal-to-noise ratio. Especially in the case of using a laser light source, the laser echoes the interference, which results in the laser output power undergoing large fluctuations. To use the LED as a light source is feasible, and the experimental result is shown in Figure 3b, while the change rate of the output power of the fiber end during outbound and backhaul is only 0.02%. So a laser light source is replaced by an LED source in the experiment, and a single fiber macro-bend is not suitable for displacement detection.

**Figure 4:**
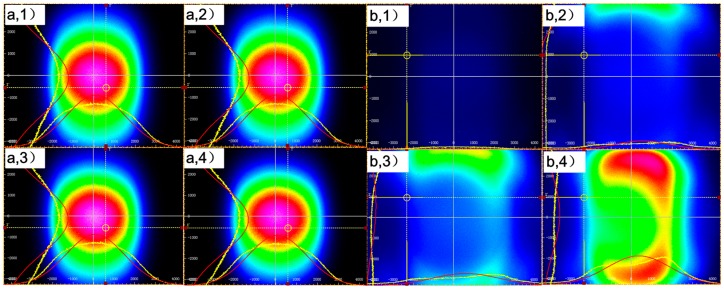
Response of the transmitting (**a**) and receiving (**b**) fiber at four different displacements: (1) d = 0 mm; (2) d = 45 mm; (3) d = 90 mm; (4) d = 135 mm.

The Beam Quality Analyzer (BC106N-VIS/M, Thorlabs) used for analyzing the energy coupling and distribution of the transmitting fiber and receiving fiber of two twisted optic fibers is also shown in Figure 4. Light intensity at the end of the transmitting fiber and the receiving fiber shown on the Beam Quality Analyzer change with the displacement. Figure 4a shows the light intensity distribution of the transmitting fiber when the displacements are 0 mm, 45 mm, 90 mm and 135 mm. 

The center to edge of the figure corresponds to the optical fiber core to cladding. The light intensity gradually weakens from the center to the edge. Figure 4a shows an output power of the transmitting fiber that can be obtained with this system. From the graph, the mode field energy of transmitting fiber mainly concentrates in the core, which is determined by the multimode fiber transmission characteristics [16,17]. The center energy changes with radius of the macro-bend on a small scale, but shifts significantly with the light source fluctuations, which means a low contribution to the macro-bend coupling effect. Therefore, when a single fiber is used for displacement detection, the signal-to-noise ratio is poor and the output power scarcely changes with displacement. 

As shown in Figure 4b, the receiving fiber retains no energy when the displacement is 0 mm, and, with enhanced displacement, the light intensity increases gradually. The mode field is distorted, and the main energy concentrates near the cladding, demonstrating that the energy is coupling from the transmitting fiber due to the macro-bend effect of two twisted fibers. This energy is modulated by the macro-bend radius, so the signal-to-noise ratio is high. There are three main reasons to explain why the light intensity distribution is irregular. The first reason is that the POFs are isotropic, but the macro-bend structure is “anisotropic”. The second reason is that the energy coupled into the receiving fiber varies since the energy changes with the different macro-bend position of the transmitting fiber. The last reason is that the complex mode field distribution of the multimode fiber, when the light field changes by the macro-bend, produces a more complex energy distribution field. Thus, the energy distribution of the receiving fiber is more complex than the transmitting fiber. 

**Figure 5:**
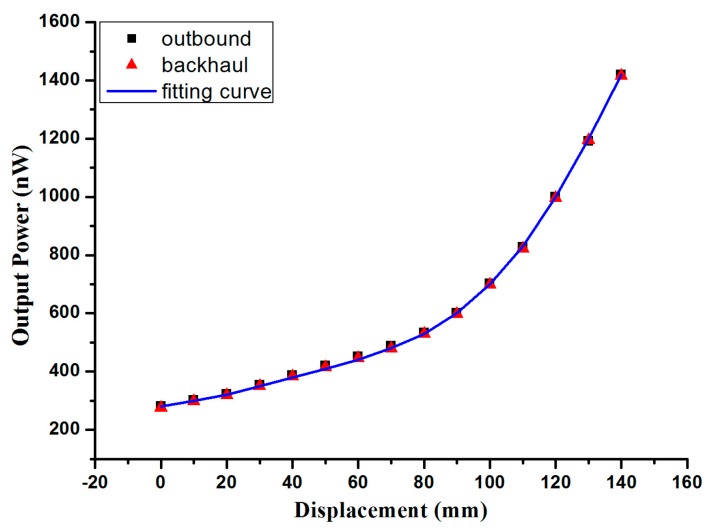
Output power of receiving fiber changes with displacement.

As in the above results, the energy of the receiving fiber changes significantly with the displacement. As plastic optical fiber is easy to bend, a twisted structure is more stable, and a two twisted optic fibers structure for displacement sensing is adopted. Figure 1 shows the schematic of the experimental setup. The free ends of two twisted fibers move forward at a constant speed, while the radius of the macro-bend increases as the fiber moves, and this process is called outbound. Then the fiber is released at a constant speed in the backhaul process with the radius of the macro-bend increasing gradually. The prototype has been completed and the result is shown in Figure 5.

The fitting curve proposed by MATLAB simulation, is

(8) $$P_{2}(d)=0.003d^{3}+0.0005d^{2}+0.979d+282.2139$$

**Figure 6:**
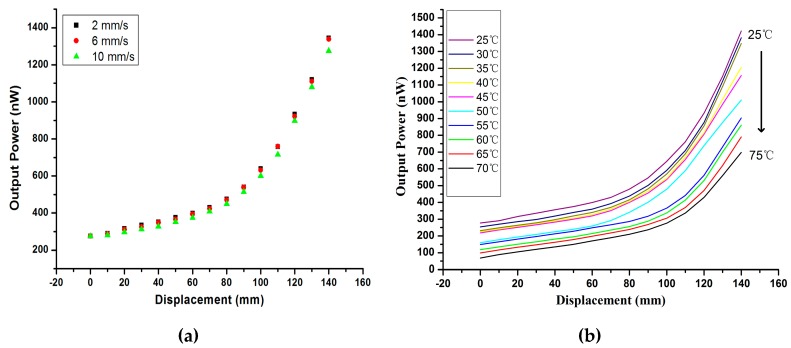
Output power of receiving fiber changes with displacement at different speeds (**a**); Output power of receiving fiber changes with displacement at different temperatures (**b**).

In the experiment, the light source power is set at 39.027 mW. Although the output power of the receiving fiber is only of nW, the resolution of the Power Meter at 1 nW is enough to be detected. The motorized stage is set to draw the free end of two twisted fibers at a constant speed of 2 mm/s, and the power meter linked to the free end of the receiving fiber is used to detect the output power of receiving fiber at the same time. From the result of Figure 5, under the displacement range of 0–140 mm, the output power of the receiving fiber increases gradually, and the output power shift is approximately linear with a displacement of 110 mm–140 mm. The sensitivity is 19.805 nW/mm; accuracy, 0.12 mm resolution, respectively. For the displacement from 140 mm to 0 mm, the output power decreases in real time, while the systematic retrace error is less than 0.05 nW/mm in the experiment. When the fiber end is drawn at different speeds of 2 mm/s, 6 mm/s, 10 mm/s, as shown in Figure 6a, the displacement-output power curve shows a slight overall decline.

Temperature is an important factor in the external environment for displacement application. As the normal operating temperature of plastic optical fiber increases up to 70 °C, the sensor is affected by temperature (from 25 °C to 70 °C) as shown in Figure 6b. With an increase of the temperature, the displacement-output power curve shows an overall decline, and the sensitivity is also decreased. Because of the rising temperature, the light performance of the plastic optical fiber gets worse, and the coupling capacity between the two twisted fibers is reduced. From the experimental results above, this system is used with displacement sensors during this development effort within a stable structure.

## 4. Conclusions

A novel optical fiber wide-range displacement sensor based on a macro-bend coupling effect has been developed, which exhibits a good robustness and low cost. According to the principle of coupling between two closely optical fibers, a two twisted fibers sensing structure is designed. A Beam Quality Analyzer was used to analyze the energy coupling and distribution of the two fibers to find out that the fiber which has no intensity originally generates a lot of light intensity, which changes with the displacement. This structure avoids the sensing signal shifts influenced by visible light and light sources and realizes the displacement measurement within a wide range of 0–140 mm. Moreover, it is effective and stable at different temperatures and speeds. The output power is approximately linear with displacement of 110 mm–140 mm under room temperature and 2 mm/s speed with 19.805 nW/mm sensitivity and 0.12 mm resolution. Compared to other sensors, the production process of this sensor is very simple, and immune to electromagnetic interference. Further work, such as compensation for the temperature change and quantitative analysis of mutual coupling of modes and interference in multimode fibers, is in progress.
